# Evaluating preferences for long term wheeze following RSV infection using TTO and best-worst scaling

**DOI:** 10.1186/1710-1492-10-S1-A64

**Published:** 2014-03-03

**Authors:** Lilla MC Roy, Nick Bansback, Carlo Marra, Roxane Carr, Mark Chilvers, Larry D Lynd

**Affiliations:** 1 Faculty of Pharmaceutical Sciences, University of British Columbia, Vancouver, Canada, V6T 1Z3; 2 School of Population and Public Health, University of British Columbia, Vancouver, Canada, V6T 1Z3; 3 British Columbia Children’s Hospital, Vancouver, Canada, V6H 3E8

## Background

Respiratory Syncytial Virus (RSV) infects 70% of infants under one year, and is a leading cause of infant bronchiolitis-related hospitalizations [1,2]. RSV is a relatively benign, temporary condition, and therefore likely to have limited impact on quality adjusted life years, however, it has potential for long-term consequences such as asthma and wheeze [3,4]. Preferences related to the trade-offs between different long and short term aspects of RSV have not been explored previously, however, deriving preferences for infant health states is very challenging as infants require proxy elicitation of preferences [5-8].

## Methods

The objectives of this study were to explore preferences surrounding attributes of RSV using proxy- and self-perspectives. Time trade-off (TTO) and best-worst scaling were used to derive utilities for health states of RSV and determine relative preferences for different levels of disease attributes. Vignettes were constructed from focus group data and expert opinion. Respondents were randomized to either a child perspective (“imagine that your child has RSV”), or an adult perspective (“imagine that you have RSV”). Experimental design for the BWS was developed using Sawtooth Software. 1000 Canadians were recruited through a market research panel facilitating a societal perspective. Five attributes were used – hospitalization status, oxygen support, presence of tubes (IV/NG), breathing symptom severity, and risk of long term wheeze. Ethics approval was obtained from the UBC BREB. Respondents completed both TTO and BWS tasks.

## Results

Disutility associated with the short term health state of RSV was significant. Disutility followed an expected gradient, with more time traded for more severe RSV, and less time traded for less severe RSV (mean range: 0.57 – 0.14). Utilities were lower in the child perspective than the adult perspective. 0% risk of long term risk of wheeze was most preferred over all other attributes, and respiratory failure was least preferred (-4.7). Strong negative preferences were similar for IV/NG (-3.3), ICU admission (-3.5), mechanical ventilation (-3.6), and severe breathing problems (-3.6). Utility associated with risk of wheeze became lower as risk increased, with a relative preference for 80% risk of wheeze (-2.8) between moderate (-1.5) and severe (-3.7) breathing problems.

## Conclusions

Preferences indicate societal willingness to accept immediate, short term, potentially clinically significant consequences to avoid long term risk of wheeze. This study provides utilities that can be utilized for the evaluation of any potential or proposed treatment of RSV in children, and is important to understanding and applying priorities in health care.
